# Lack of recovery of the long-spined sea urchin *Diadema antillarum* Philippi in Puerto Rico 30 years after the Caribbean-wide mass mortality

**DOI:** 10.7717/peerj.8428

**Published:** 2020-02-12

**Authors:** Evan Tuohy, Christina Wade, Ernesto Weil

**Affiliations:** Department of Marine Science, Universidad de Puerto Rico, Recinto de Mayagüez, Mayagüez, Puerto Rico, Puerto Rico

**Keywords:** *Diadema antillarum*, Mass mortality, Population status, Puerto Rico

## Abstract

Caribbean populations of the long-spined black sea urchin *Diadema antillarum* Philippi were decimated by a disease-induced mass mortality in the early 1980’s. The present study provides an updated status of the *D. antillarum* recovery and population characteristics in La Parguera Natural Reserve, Puerto Rico. The last detailed study to assess population recovery in 2001, indicated a slow, and modest recovery, albeit densities remained far below pre-mass mortality levels. Population densities were assessed along three depth intervals in six reef localities and one depth in three lagoonal sea-grass mounds using ten 20 m^2^ (10 × 2 m) belt-transects at each depth interval. Most of these were previously surveyed in 2001. All individuals encountered along the belt transects were sized *in situ* with calipers and rulers to characterize the size (age) structure of each population and get insight into the urchin’s population dynamics and differences across localities in the area. Habitat complexity (rugosity) was assessed in all depth intervals. No significant differences in population densities between reef zones (inner shelf and mid-shelf) were observed, but significantly higher densities were found on shallow habitats (<5 m depth; 2.56 ± 1.6 ind/m^2^) compared to intermediate (7–12 m; 0.47 ± 0.8 ind/m^2^) and deep (>12 m; 0.04 ± 0.08 ind/m^2^) reef habitats in almost all sites surveyed. Habitat complexity had the greatest effect on population densities at all levels (site, zone and depth) with more rugose environments containing significantly higher densities and wider size structures. Comparison between survey years revealed that *D. antillarum* populations have not increased since 2001, and urchins seem to prefer shallower, more complex and productive areas of the reef. Populations were dominated by medium to large (5–9 cm in test diameter) individuals and size-frequency distributions indicated that smaller juveniles were virtually absent compared to 2001, which could reflect a recruitment-limited population and explain in part, the lack of increase in population densities. The limited temporal scale of this study, high horizontal movement of individuals along the complex, shallower reef and inshore habitats could also explain the general decline in mean densities. Other extrinsic factors affecting reproductive output and/or succesful recruitment and survival of juveniles likely contribute to the high variablility in population densities observed over time.

## Introduction

Population recovery of the Caribbean long-spined sea urchin, *Diadema antillarum* (Philippi, 1845) after the widespread, species-specific epizootic event that nearly eradicated the species in the early 1980s, has been variable but slow (Randall, Schroeder & Starck II, 1964; Ogden & Lobel, 1978; Lessios et al., 1984; Liddell & Ohlhorst, 1986; Hughes, Reed & Boyle, 1987; Hughes, 1994; Aronson & Precht, 2000; Chiappone et al., 2002; Weil, Torres & Ashton, 2005; Lessios, 2005; Lessios, 2016). Prior to the mass mortality event, *D. antillarum* was a ubiquitous keystone grazer and bioeroder with high densities in the wider Caribbean (Randall, Schroeder & Starck II, 1964; Hunter, 1977; Ogden, 1977; Sammarco, 1982; Weil, Losada & Bone, 1984; Hay & Taylor, 1985; Carpenter, 1986; Lessios, 2005) reaching densities of 71 ind/m^2^ (Sammarco, 1980); however, such high natural densities are optimal for virulent diseases to spread rapidly due to abundant and densely packed hosts that facilitates contagion and fast spread of disease (Lafferty & Hofmann, 2016).

The epizootic event that started in Panama in January 1983 and spread over 3.5 million km^2^ in one year (Lessios et al., 1984) produced 87–100% mortality in Panama, Curaçao, Barbados, Jamaica and many other localities (Lessios et al., 1984; Lessios, Robertson & Cubit, 1984; Bak, Carpay & De Ruytervan Steveninck, 1984; Hughes et al., 1985; Hunte, Côté & Tomascik, 1986). Together with a concurrent coral white band disease (WBD) episode that wiped out up to 99% of the populations of the foundational coral genus *Acropora* across their geographic distribution (Aronson & Precht, 2001; Weil & Rogers, 2011), resulted in, by far, the worst invertebrate disease-induced mass mortalities in recent times (Weil & Rogers, 2011). Shortly thereafter, a phase-shift from coral-dominated to algal-dominated reefs with detrimental effects to coral populations and reef health was reported for many overfished localities throughout the region (Bak, Carpay & De Ruytervan Steveninck, 1984; Hay & Taylor, 1985; Liddell & Ohlhorst, 1986; Hughes, Reed & Boyle, 1987; Hughes, 1994). The dramatic increase in algal cover was most notable in areas like Jamaica, where overfishing had significantly reduced the number of herbivorous fish which increased importance on grazing by *D. antillarum*. However, this was not always the common pattern throughout the Caribbean, especially in areas where populations of herbivorous fish were not overfished (Hay, 1984; Liddell & Ohlhorst, 1986; Mumby et al., 2006; E Weil, pers. obs., 1985). Disease outbreaks and bleaching events continued impacting Caribbean reefs through the 1990’s and 2000’s, killing coral and other key invertebrate foundational species and opening more space for algae to grow (Miller et al., 2006; Wilkinson, 2006; Weil et al., 2006; Rogers et al., 2008; Weil et al., 2009; Eakin et al., 2010).

Over the last 30 years, many reef areas of the Caribbean have experienced modest *D. antillarum* population recovery (Chiappone et al., 2002; Chiappone et al., 2010; Lessios, 2005; Miller et al., 2003; Weil, Torres & Ashton, 2005; Debrot & Nagelkerken, 2006). Barbados (Hunte & Younglao, 1988) and Jamaica (Edmunds & Carpenter, 2001) were among the first areas to show clear signs of population recovery, although these observations may result from variable levels of mortality (susceptibility differences) and/or sampling effort, and usually reflect a short time period. Unlike Barbados and Jamaica, population recovery has been low to modest in most other localities and remains especially low in the Florida Keys (Chiappone et al., 2002; Chiappone et al., 2010; Miller et al., 2009; Lazar et al., 2005). However, increases in recruitment rates reported in St. Croix (Miller et al., 2003) and Curaçao (Vermeij et al., 2010) indicate that populations are starting to recover in other reefs localities around the Caribbean.

Recovery is probably denso-dependent as shown by Miller et al. (2007), who found that juvenile persistence was significantly higher in treatments of higher adult density. Caribbean-wide, it is important to monitor population recovery to assess overall reef health. In southwest Puerto Rico, the first signs of modest population recovery were observed approximately 17 years after the mass mortality event; however, densities still remained far below those recorded for the region prior to the die-off (Weil, Torres & Ashton, 2005). The main objective of this study was to assess the status of *D. antillarum* populations 30 years after the mass mortality event by examining urchin abundances, spatial distribution and population size structure, and testing any potential relationship with habitat characteristics along a nearshore to offshore gradient in La Parguera Natural Reserve (LPNR), southwest coast of Puerto Rico. The second objective was to compare results with those of reported for 2001 and with other Caribbean localities.

## Materials & Methods

### Study area

**Figure 1:**
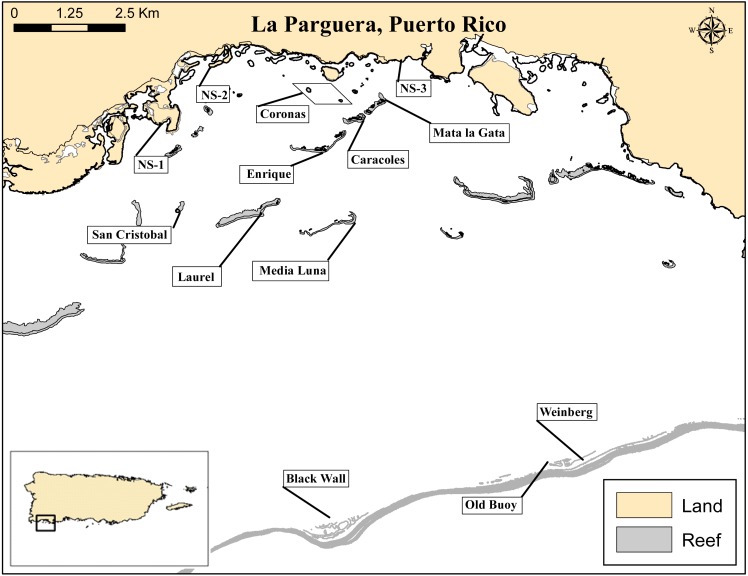
La Parguera Natural Reserve, Puerto Rico. Map of nearshore (NS-1-3), seagrass mounds (Coronas) and reef localities sampled. Exploratory surveys were conducted at nearshore (NS-1-3) and deep reef localities (Weinberg, Old Buoy and Black Wall). Inset shows relative location of La Parguera in relation to the island of Puerto Rico.

This study was conducted during the fall months of 2013 in the La Parguera Natural Reserve along the southwest coast of Puerto Rico (Fig. 1). This area is characterized by a broad, gradually sloping insular shelf extending approximately 10 km offshore before reaching the shelf break at 25–35 m depth. The insular shelf supports extensive mangrove forest fringing the main coast and on coral keys, seagrass and coral reef development of high ecological, economic and cultural value (Pittman et al., 2010). Coral reefs are distributed across an inshore-offshore gradient into three main zones; the inner shelf reefs, mid-shelf reefs and outer shelf reef zones. Inner shelf and mid-shelf reef zones are formed mostly by long fringing reefs along coral cays and platform patch reefs (0–18 m depth) approximately 0.5–4 km from shore. Shelf-edge or outer reefs are offshore, deep, spur-and-groove reefs (18–35 m depth) extending to the shelf break (Weil, Cróquer & Urreiztieta, 2009; Hubbard et al., 2008).

### Sampling locations

A total of six fringing reef sites, from two reef zones (inner and mid-shelf), and three lagoonal seagrass-covered mounds (coronas) were surveyed to assess the population density, size structure, and habitat complexity/rugosity associated with *D. antillarum* populations (Fig. 1 and Table 1). Additionally, to assess the spatial extent of *D. antillarium* distributions, exploratory surveys were conducted along three nearshore rocky and mangrove habitats and observations were made at three deep reef sites (Fig. 1). Quantitative surveys of urchin populations in the reef localities took place along the fore reef habitats of the coral cays. Three of the reefs were inner shelf reefs (1–2 km from shore; Enrique, Caracoles and Mata la Gata), and three were mid-shelf reefs (3–4 km from shore; Media Luna, Laurel and San Cristobal). The three coronas, were approximately 0.5 km from shore, *Thalassia* dominated, oval-shaped and approximately the same size (900 m^2^), with depths ranging between 0–10 m. Previous reports indicate that dense population of *Diadema* used to inhabit these seagrass mounds (Weil, Torres & Ashton, 2005).

**Table 1:** List of habitats and reefs surveyed for *Diadema antillarum* and their habitat type, characteristics and depth range (m). Data are habitat type, characteristics and depth range (m).

**Site**	**Habitat type**	**Characteristics**	**Depth range (m)**
Corona 1	Seagrass	Lagoonal mound	0–3
Corona 2	Seagrass	Lagoonal mound	0–3
Corona 3	Seagrass	Lagoonal mound	0–3
Caracoles	Reef	Inshore: Protected fringing reef	0–15
Enrique	Reef	Inshore: Protected fringing reef	0–18
Mata la Gata	Reef	Inshore: Protected fringing reef	0–18
Laurel	Reef	Midshelf: Exposed fringing reef	0–18
Media Luna	Reef	Midshelf: Exposed fringing reef	0–18
San Cristobal	Reef	Midshelf: Exposed fringing reef	0–15

### Population densities

The population densities of *D. antillarum* were estimated using ten 20 m^2^ (10 × 2 m) belt-transects in each of three depth interval habitats (0–5, 7–12, and >12 m) in each locality. Every urchin within the belt transect was counted and divers took special care to thoroughly inspect the holes and crevices of the complex habitat structure so that hidden individuals were not overlooked. Urchin average population densities and standard deviation were calculated for each habitat depth interval (average of the ten band-transects) and then, for the entire reef locality (*n* = 30). At nearshore rocky and mangrove habitats, ten linear transects were placed parallel to the shore or mangrove roots. At each of the coronas, ten transects were laid parallel to each other in 5 m increments across the seagrass-covered mounds. The total area surveyed for both the coronas and nearshore and mangrove habitats was 200 m^2^ per location. Ten transects at each depth interval yielded a total of 30 transects per location and an area surveyed of 600 m^2^ per reef.

### Size structure

Size structure was assessed at all locations and depth intervals by measuring the oral test diameters of at least 100 individuals encountered along all the belt transects. Urchins were collected carefully along each transect, making sure that small crevices and holes were checked for presence of juveniles. Salad tongs and a prying tool were carefully used to get them out of reef recesses, placed in weighted plastic baskets and moved to adjacent sand patches where their oral test diameters were measured using a plastic ruler. Care was taken to assure that all urchins were returned to the same habitat unharmed (Weil, Torres & Ashton, 2005).

### Habitat complexity/rugosity

The rugosity along each belt-transect was estimated using the “chain-and-tape” method outlined by Risk (1972) and McCormick (1994). A chain, marked with cable ties at 10 cm increments, was carefully draped over the substrate along the center of each belt-transect. A rugosity index was calculated as the ratio of the length of the chain (A) draped over the reef profile to the straight linear distance of the transect (*B* = 10 m), where R = A/B.

### Data analysis

To assess the current status of *D. antillarum* populations in the LPNR we used a total of 210 belt-transects from nine sites: three seagrass mounds (one depth*10 per depth; *n* = 30), three inner-shelf reefs (three depths*10 per depth; *n* = 90) and three mid-shelf reefs (three depths*10 per depth; *n* = 90). Differences in population densities of *D. antillarum* were analyzed using permutational multivariate analysis of variance (PERMANOVA) with the Primer v7 software (Clarke & Gorley, 2015). Two PERMANOVA tests were run using Euclidian distances among samples since the test was used on a univariate measure (density).

The first PERMANOVA was used to identify differences in population densities between reef zones, depth interval and rugosity. The PERMANOVA model considered the effects of the factors of reef zone (fixed factor, two levels: inshore and mid-shelf), reef site (random factor, six levels nested within zone: inshore = Mata la Gata, Caracoles, Enrique; and mid-shelf = Media Luna, Laurel and San Cristobal), depth (fixed factor, three levels: 0–5 m, 7–12 m, and >12 m), with rugosity considered as a co-variable. Reef zone was a fixed factor that test for potential differences between two distinct ecological regions in the La Parguera Natural Reserve where differences in other assemblages (fish and corals) have been observed. Reef was a random factor that estimates natural variation within each zone and is not part of any specific hypothesis. Depth was a fixed factor included to test for the well-known gradient effect of species richness and diversity with depth. The three main factors described above resulted in two first order interactions: (1) Zone*Depth indicates that the effect of depth depends on zone; and (2) Site(Zone)*Depth indicates that the effect of depth depends on the site nested within the zone.

To assess changes in population densities over time, the results of this study were compared to previous surveys conducted in 2001 at seven sites: three seagrass mounds, two inner-shelf reefs and three mid-shelf reefs (Weil, Torres & Ashton, 2005). The second PERMANOVA was used to identify differences in density of *D. antillarum* between the two sampling years (2001 and 2013). Differences in the experimental design between sampling years did not allow for direct comparison of data sets using the linear model described above. For this reason, the following factors were considered: years (fixed factor, two levels: 2001 and 2013), reef zone (fixed factor, two levels: inshore and midshelf) and reef site (random factor, four levels nested within zone: inshore = Caracoles and Enrique; and midshelf = Media Luna and Laurel). Sampling year is orthogonal with reef zone and site.

Test diameter was used to compare the size structure of *D. antillarum* populations between reef zones, reef localities and depth intervals.

## Results

### Population densities and distributions

**Figure 2:**
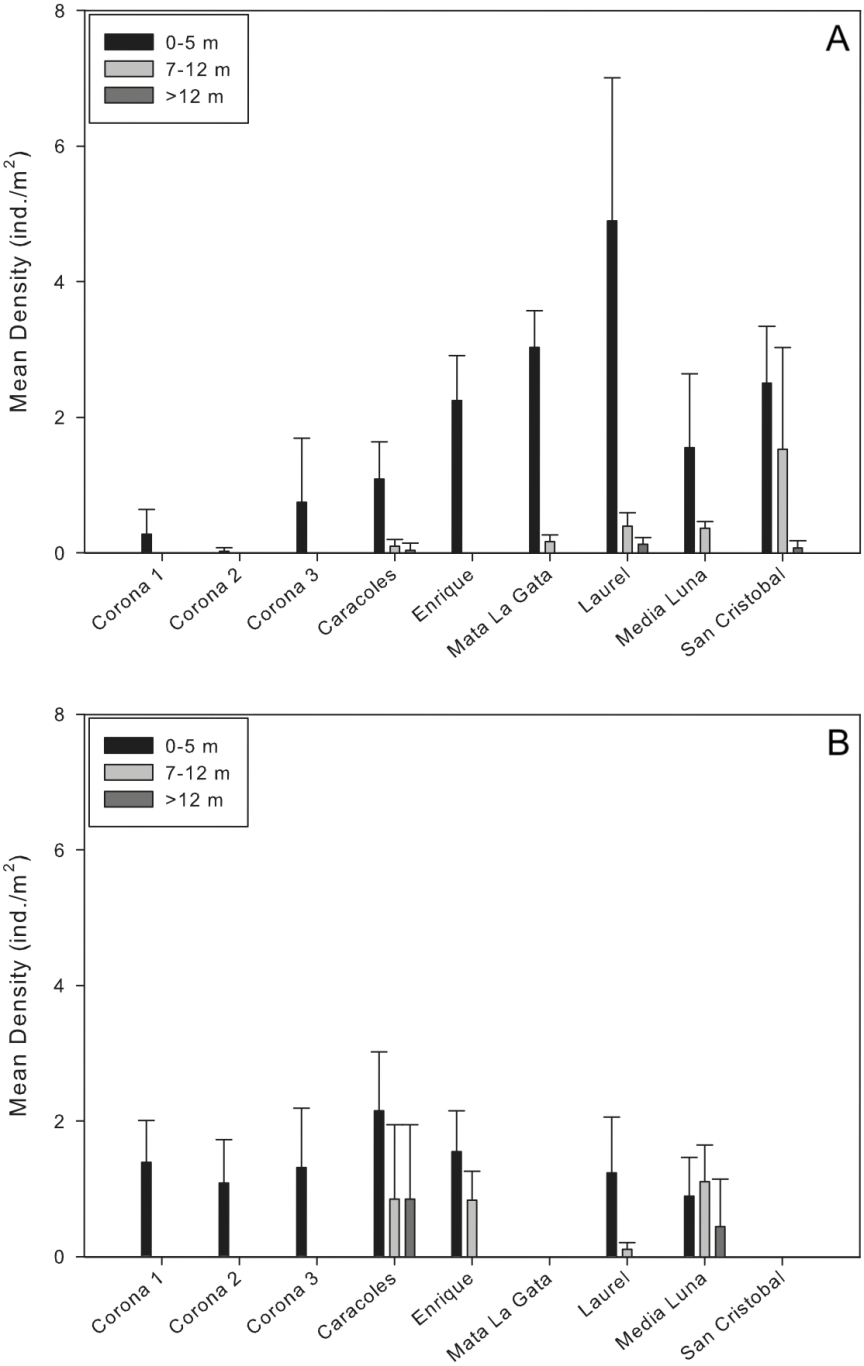
Comparison of *Diadema antillarum* densities between years and sampling localities. Data are mean densities (±SD) of *D. antillarum* in (A) each of the reefs and seagrass mounds sampled in this study and (B) each of the reef and seagrass mounds sampled in 2001 (Weil, Torres & Ashton, 2005). Mata La Gata and San Cristobal were not sampled in the 2001 study. The number of transects completed at each depth interval in the 2001 study are as follows: Corona 1, *n* = 8; Corona 2, *n* = 7; Corona 3, *n* = 5; Caracoles, *n* = 12 (0–5 m), *n* = 6 (7–12 m), *n* = 6 (>12 m); Enrigue, *n* = 6 (0–5 m), *n* = 6 (7–12 m), *n* = 0 (>12 m); Laurel, *n* = 8 (0–5 m), *n* = 4 (7–12 m), *n* = 0 (>12 m); Media Luna, *n* = 12 (0–5 m), *n* = 6 (7–12 m), *n* = 6 (>12 m). All data are individuals per m^2^.

Mean population densities at all sites ranged from 0.02 ± 0.05 ind/m^2^ at a corona to 1.8 ± 2.52 ind/m^2^ at Laurel reef (Table 2; Fig. 2A). Specifically at the coronas, mean population densities ranged from 0.02 ± 0.05 to 0.7 ± 0.89 ind/m^2^ while at reef localities mean population densities ranged from 0.44 ± 0.58 ind/m^2^ (Caracoles reef) to 1.8 ± 2.52 ind/m^2^ (Laurel reef; Fig. 2A). In all instances, there were no *D. antillarum* encountered during exploratory surveys of the near-shore and deep reef locations. The highest urchin densities observed during surveys were along the shallow transects (<5 m) at Laurel reef with 4.9 ± 2.1 ind./m^2^ (Table 2).

**Table 2:** Mean population densities of *Diadema antillarum* and reef complexity (rugosity measurements) recorded at each sampling depth (shallow, intermediate and deep) for all sampling sites where urchins were observed. Data are site, sampling depth (m), mean density (ind./m^2^) and mean rugosity.

**Site**	**Samplingdepth (m)**	**Mean density(ind./m2)**	**Meanrugosity**
Corona 1	0–3	0.28 ± 0.48	1.0 ± 0
Corona 2	0–3	0.02 ± 0.05	1.0 ± 0
Corona 3	0–3	0.74 ± 0.95	1.0 ± 0
	Total	0.35 ± 0.45	1.0 ± 0
Caracoles	0–5	1.09 ± 0.5	1.5 ± 0.14
	7–12	0.1 ± 0.1	1.3 ± 0.13
	>12	0.04 ± 0.1	1.3 ± 0.11
	Total	0.41 ± 0.25	1.37 ± 0.13
Enrique	0–5	2.25 ± 0.66	1.6 ± 0.07
	7–12	0.01 ± 0	1.3 ± 0.07
	12 +	0	1.1 ± 0.08
	Total	0.75 ± 0.22	1.33 ± 0.73
Mata la Gata	0–5	3.03 ± 0.54	1.3 ± 0.1
	7–12	0.2 ± 0.1	1.3 ± 0.12
	>12	0.01 ± 0	1.4 ± 0.17
	Total	1.07 ± 0.21	1.33 ± 0.13
Laurel	0–5	4.9 ± 2.11	1.7 ± 0.11
	7–12	0.4 ± 0.2	1.5 ± 0.04
	>12	0.13 ± 0.1	1.3 ± 0.1
	Total	1.81 ± 0.8	1.5 ± 0.25
Media Luna	0–5	1.56 ± 1.09	1.5 ± 0.09
	7–12	0.37 ± 0.1	1.3 ± 0.05
	>12	0	1.1 ± 0.07
	Total	0.64 ± 0.4	1.3 ± 0.21
San Cristobal	0–5	2.51 ± 0.84	1.4 ± 0.11
	7–12	1.53 ± 1.5	1.3 ± 0.03
	>12	0.08 ± 0.1	1.1 ± 0.06
	Total	1.37 ± 0.81	1.27 ± 0.07

Results of the permutational analysis of variance (PERMANOVA) showed that there was a significant difference in population densities of *D. antillarum* within reef sites sampled (*df* = 4, Pseudo-F = 13.05, *p* = 0.00; Table 3), however, no differences were observed in mean population densities between reef zones (inner shelf and mid-shelf; *df* = 1, Pseudo-F = 0.64, *p* > 0.05; Table 3). Patterns of *D. antillarum* population density, in terms of depth, were distinctly different. When comparing all shallow sites sampled (reefs and coronas), there were significant interactions between population densities and depth at the site level, with differences observed within all shallow sites (*df* = 8, Pseudo-F = 22.40, *p* = 0.00; Fig. 2A and Table 3). The same was observed when comparing densities between depth intervals at reefs sites, with shallow habitats (0–5 m) having significantly higher densities compared to intermediate and deep habitats (*df* = 2, Pseudo-F = 8.68, *p* = 0.01; Table 3). This was apparent at all reef sites except San Cristobal, where no differences in population density between shallow and the intermediate depth habitat was observed (Fig. 2A).

**Table 3:** Results of the permutational analysis of variance (PERMANOVA) of difference in *D. antillarum* population density reef zones, depth and rugosity. Sampled reef sites were nested within zone.

**Factor**	**Df**	**SS**	**MS**	**Psuedo-F**	**P(perm)**
Zone	1	4.0983	4.10	0.64	0.446
Depth	2	189.63	94.82	8.68	0.009
Rugosity	1	8.5975	8.60	7.40	0.007
Site(Zone)	4	25.195	6.30	13.05	0.000
Zone*Depth	2	14.384	7.19	0.67	0.542
Site(Zone)*Depth	8	86.502	10.81	22.40	0.000

### Size structure

**Figure 3:**
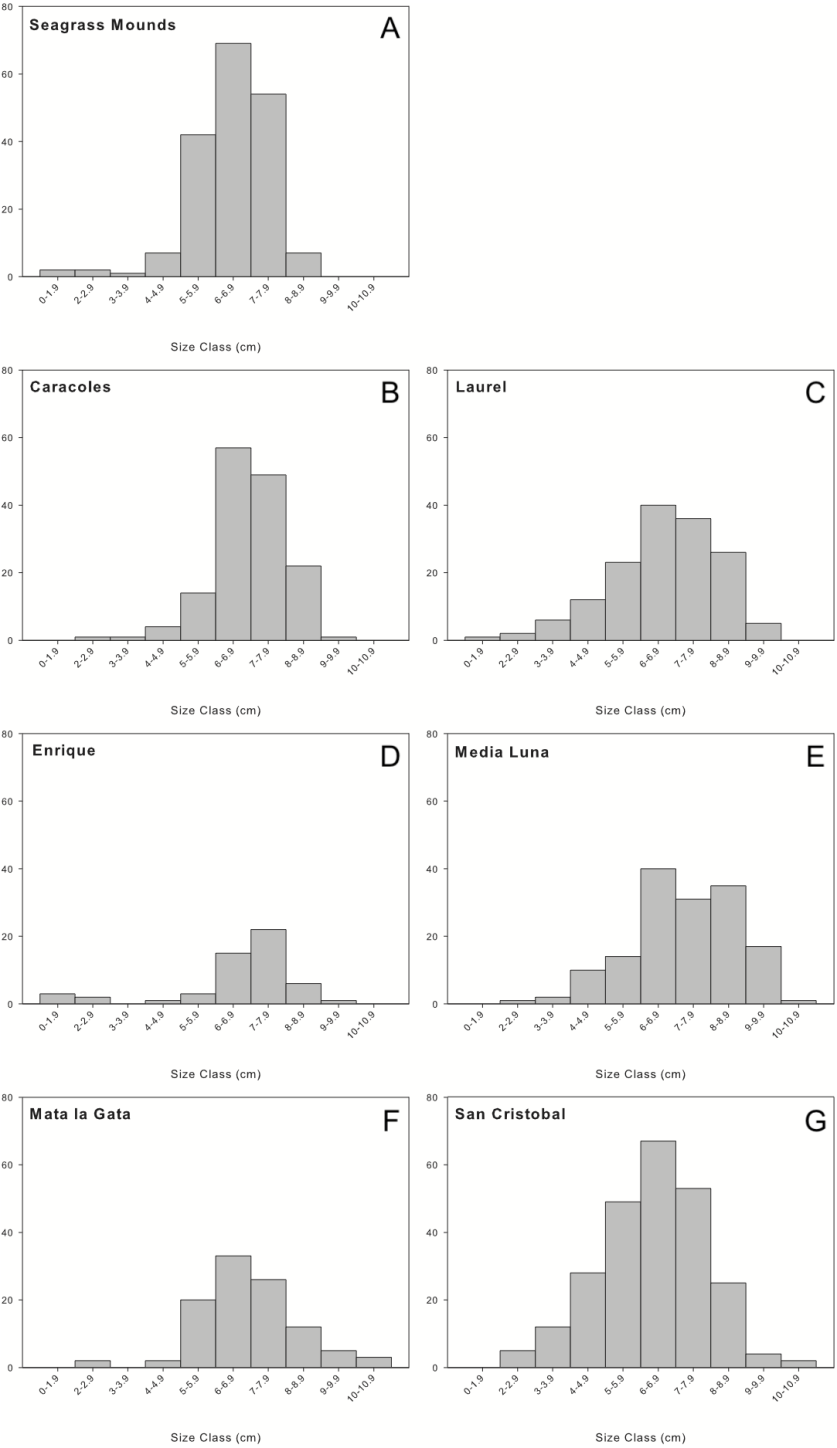
Size frequency distributions of *Diadema antillarum* observed at each sampling location. Data are oral test diameter (cm) of *D. antillarum* collected and measured at (A) seagrass mounds, (B) Caracoles reef, (C) Laurel reef), (D) Enrique reef, (E) Media Luna reef, (F) Mata la Gata reef, and (G) San Cristobal reef.

A total of 1,036 individuals were collected and measured from all sampling locations. Overall, urchin populations were dominated by medium to large individuals (5–9 cm in test diameter; Fig. 3). Populations in seagrass mounds had mostly medium size urchins ranging from 5.68 ± 1.21 to 6.4 ± 0.88 cm, however, slightly larger sizes were encountered for reef populations, with test diameters ranging from 6.38 ± 1.76 to 6.95 ± 1.51. Although size distributions did not vary between reef zones, reef sites or depth intervals, mid-shelf reefs contained the widest range of size classes, with the majority of individuals in the 5 to 9 cm size range. Laurel and San Cristobal showed almost normal distributions with higher abundances of medium size classes (>4 cm) (Fig. 3). Similar trends were observed in 2001 with populations dominated by medium to large individuals (6–9 cm) and very few juveniles (Weil, Torres & Ashton, 2005).

### Habitat complexity

**Figure 4:**
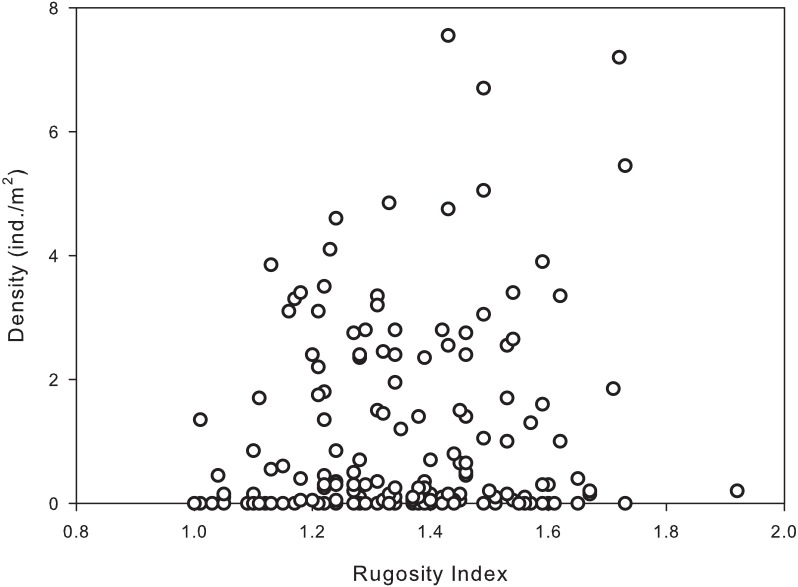
Significant correlation between population densities of *Diadema antillarum* and habitat complexity (rugosity) were found at each reef site (Spearman rank correlation: *r*_*s*_ = 0.178, *p* < 0.05). Data are the population density (ind./m^2^) and rugosity index recorded from transects at reef sites (*n* = 180).

Habitat complexity ranged from very low (flat pavement) to high (shallow reefs dominated by dead and living *Acropora palmata* and/or *A. palmata* rubble). The majority of reef sites sampled had intermediate to high rugosity indices (1.3–1.8), with the highest urchin population densities occurring in the more complex habitats/reefs. Results of the permutational analysis of variance (PERMANOVA) indicated that rugosity had a significant effect on population density at all levels (zone, site and depth) (*df* = 1, Pseudo-F = 7.40, *p* = 0.01; Table 4). A Spearman’s rank correlation indicated that there was a significant positive association between increased rugosity and population densities of *D. antillarum* (*r*_*s*_ = 0.178, *p* < 0.05; Fig. 4), however, there was a weak relationship between the two variables.

### Comparison of densities between sampling years (2001 and 2013)

Mean densities of *D. antillarum* between sampling sites and years ranged from 0.83–1.55 ind/m^2^ in 2001 to 0.02–1.8 ind/m^2^ in 2013 (Fig. 2). For each reef site sampled, *D. antillarum* population densities in shallow habitats were higher in 2013 compared to densities reported in 2001, with the exception of Caracoles (Fig. 2). Conversely, urchin population densities were higher in the intermediate and deep reef habitats of all but one reef (Laurel) in 2001 compared to 2013 (Fig. 2). Limited number of replicates prohibited comparison of population densities between sampling years directly with PERMANOVA. However, by separating the factors of zone and year within the PERMANOVA analysis, comparing sampling year and zones indicated that there was a significant difference in population densities between coronas and inner shelf reefs with higher densities encountered in 2001 (*df* = 2, Pseudo-F = 5.52 , *p* = 0.00; Table 4), and no significant differences for midshelf reefs between 2001 and 2013 (Fig. 2).

**Table 4:** Results of the permutational analysis of variance (PERMANOVA) of difference in *D. antillarum* population density between sampling years.

**Factor**	**Df**	**SS**	**MS**	**Psuedo-F**	**P(perm)**
Year	1	12.15	12.15	8.42	0.00
Zone	2	3.21	1.60	0.41	0.69
Site(Zone)	4	15.73	3.93	2.72	0.03
Year*Zone	2	15.95	7.98	5.52	0.00

## Discussion

Results of this study indicate that populations of the black sea urchin *D. antillarum* show no significant increase or decrease in population densities in La Parguera Natural Reserve since the last population assessment 12 years prior (Weil, Torres & Ashton, 2005). *D. antillarum* were patchily distributed across the lagoonal seagrass mounds and fringing reefs, with mean population densities ranging from 0.27 ± 0.59 to 1.81 ± 2.52 ind/m^2^, with significant variation between reefs sampled. Population densities on reef sites surveyed ranged from 0.41 ± 0.25 to 1.81 ± 2.52 with no differences between the inner shelf and mid-shelf reef zones. No urchins were encountered during exploratory surveys conducted along nearshore rocky or mangrove or outer shelf habitats. Analysis of shallow habitats where urchins were observed in highest densities (reefs and coronas <5 m depth) revealed significantly higher densities of urchins found on the reef habitats compared to coronas, indicating that although urchins do persist in these shallow lagoonal habitats, they experience enhanced recruitment and higher survivorship on shallow reefs.

Depth seemed to influence distribution and densities of *D. antillarum* in the reefs sampled, with significantly higher population densities on shallow habitats compared to deeper habitats. This is mostly attributable to the herbivorous feeding habits of *D. antillarum* and the higher food availability in shallower areas (Lewis, 1964; Randall, Schroeder & Starck II, 1964; Sammarco, 1980; Weil, Losada & Bone, 1984). Population densities in shallow regions ranged from 1.09 ± 0.55 to 4.9 ± 2.11 ind/m^2^, which in most cases were levels of magnitude higher than those encountered along deeper depth intervals at reefs. Hydrodynamics has been shown to influence the vertical distribution of urchins along reef tracts, with low wave energy environments commonly exhibiting higher urchin densities (Tuya et al., 2007; Clemente & Hernández, 2008). The insular shelf of La Parguera is primarily considered a low-wave energy environment, allowing for high *D. antillarum* densities to occur along the well-lit, shallow forereef platforms, where productivity is high, and algae plentiful.

Habitat complexity had the greatest influence on *D. antillarum* population on all levels. Historically, habitat complexity has proven to be a major factor influencing urchin distributions, as habitat complexity provides refuge from predation (Weil, Losada & Bone, 1984; Lee, 2006; Clemente & Hernández, 2008), however, even though significant interactions exist, the results of this study indicate a weak relationship between the variables. This is likely due to the low densities and low spatial/temporal variability in densities encountered in the area. Furthermore, the overall lack of large predatory fish species capable of feeding on adult *D. antillarum* (Garcia-Sais et al., 2005; Vermeij et al., 2010), competition for refuge habitats and higher availability of algae in open flat areas in the shallow reefs are likely allowing urchins to move into less rugose habitats. The absence of *D. antillarum* on deeper transects reveals that urchin are primarily confined to shallow, highly productive regions of the reef, therefore, rugosity is becoming less of a factor influencing populations on reefs in the area.

Size frequency distributions at most reef sites and all lagoonal seagrass mounds showed unimodal distributions with a persistence of medium to large individuals and an absence of juveniles within all localities. Laurel and San Cristobal showed almost normal distributions with higher abundances of smaller size classes (>4 cm), indicating a stable population with higher settlement and recruitment rates of smaller individuals, compared to other reefs sampled. Weil, Torres & Ashton (2005) reported high abundances of juveniles along back reef lagoon- and semi-exposed shallow habitats, but this was not the case in the current study, as there were no urchins encountered on survey of these habitats.

An overall absence of small individuals with an increase in the number of larger individuals could suggest episodic recruitment facilitated by the chance convergence of oceanographic conditions that support settlement (Lessios, 1995). This has the obvious benefit to population dynamics as increases in the occurrence of larger individuals are characterized by higher fecundity and enhances post-settlement success, which will help with the repopulation of *Diadema* in many localities (Miller et al., 2009). Additionally, size frequencies skewed to larger individuals suggests that individuals that successfully settle have high survivorship.

Since there were very few small individuals observed during surveys, there is most likely high mortality occurring in the pre-settlement or early post-settlement stage, with survivorship increasing as individuals pass this threshold (Vermeij et al., 2010). This may be explained by the shift in reef fish community structure that has occurred in La Parguera over the past several decades. The area is typical to that of most overfished areas, with an overall lack of abundances of large-bodied predatory fish species, giving rise to abundances skewed to smaller planktivores and herbivores spp. (Garcia-Sais et al., 2005), all of which have the potential of negatively impacting survival rates of pre- and early post-settling larvae of *D. antillarum*, by either feeding directly on pre-settlement planktonic larvae or indirectly consuming early post-settlers while grazing the benthos (Vermeij et al., 2010). The three-dimensional structure and the hiding behavior of juveniles may influence survey results; however, efforts were made to closely examine holes and crevices for the juveniles. An alternative for future surveys is to conduct surveys at crepuscular periods when juveniles are more actively grazing.

The current study varied slightly to previous work that report the population status of *D. antillarum* in the area, but in ways that make the current study more robust. Weil, Torres & Ashton (2005) conducted surveys at four reefs and three seagrass mounds, where the number of belt transects conducted varied among sites and depth intervals between reef sites (total area surveyed = 1,760 m^2^; total urchins sampled = 2,196). This study incorporated a standardized approach to ensure that the same area was sampled per site and per depth interval, yielding a total surface area sampled of 600 m^2^ per reef site and a total of 200 m^2^ at lagoonal seagrass mounds (total area surveyed = 5,400 m^2^; total urchins sampled = 1,036). Comparing the results from both studies revealed that overall mean densities are lower compared to the previous analyses, and this can be attributed to a distinct shift in the vertical zonation of the population. In the previous study, *D. antillarum* were more widely distributed throughout the various depth intervals (Fig. 2). In the present study, the virtual absence of *D. antillarum* deeper than 5 m, indicates that these urchins may be experiencing the same compression of depth zonation reported for other benthic marine organisms in the region (Larsen & Webb, 2009).

Over the past several decades the water quality in the La Parguera Natural reserve has deteriorated due to increased coastal deforestation for resort development and housing causing increased sedimentation and nutrient input into the marine ecosystem (Warne, Webb & Larsen, 2005; Ryan et al., 2008; Pittman et al., 2010). Sediment sampling has shown an overall doubling of marine sedimentation on the coral reef ecosystem in La Parguera over the last century (Ryan et al., 2008). Marine sediments collected from these environments showed that heavy metal concentration and total suspended solids were significantly higher in the vicinity of developed areas. Additionally, many locations sampled exhibited heavy metal concentrations exceeding reported values that cause impairment to biological systems (Hertler et al., 2009). Consequently, deterioration in water quality has been reported as a primary threat to the near shore coral reef ecosystems in the regions (Garcia-Sais et al., 2005; Pittman et al., 2010). Increase turbidity along the insular shelf of southwest Puerto Rico has had a strong influence on the benthic community structure of the region, causing significant environmental change and has resulted in a compression of coral depth zonation (Acevedo, Morelock & Olivieri, 1989).

Reef sites located further from the town of La Parguera (area of highest coastal development) are characterized with diminished land-based sedimentation, turbidity, nutrients and pollution (Hertler et al., 2009; Oliver et al., 2014). Results presented in this study indicate that the same compression of depth zonation may be occurring with population of *D. antillarum,* in all reefs except those furthest from the area of highest coastal development. Additionally, reef sites furthest from areas of high development showed increased levels of recruitment, with higher abundances of smaller individuals compared to reefs closer to town center (Fig. 4).

Although initial reports indicated a slow to modest recovery of populations in the region (Weil, Torres & Ashton, 2005), these densities were far below that of pre-mortality estimates. The most recent evaluation of population densities indicate that the long-spined urchin populations have decreased slightly since estimates completed twelve years prior. Although densities in this region exceed reports from other Caribbean islands (Lessios, 2016), it appears that these population are recruitment limited in reef areas and confined to shallower areas of the reef maybe due to decreased water quality from land-based sources. Population recovery is likely being affected by a wide range of factors including low reproductive output (i.e., low larval supply) due to low densities and restricted distribution, low recruitment, lack of grazed substrate suitable for settlement, and high post-settlement mortality (Lessios, 1995; Bak & Nieuwland 1995; Miller et al., 2009). This could be discouraging given the well documented ecological benefits to coral reefs provided by moderate densities of *D. antillarum* (Randall, Schroeder & Starck II, 1964; Ogden & Lobel, 1978; Liddell & Ohlhorst, 1986; Hughes, Reed & Boyle, 1987; Hughes, 1994; Aronson & Precht, 2000; Edmunds & Carpenter, 2001; Weil, Torres & Ashton, 2005; Lessios, 2005; Carpenter & Edmunds, 2006; Idjadi, Haring & Precht, 2010; Clemente & Hernández, 2008; Hernández et al., 2008; Chiappone et al., 2010). However, we need to keep in mind that this is a limited temporal and spatial study with only a few representative localities, therefore, results represent the status at one point in time. Given that *Diadema* densities are overall low, so intraspecific competition for refuges and food is low, and the capacity for this species to move across reef habitats and other coastal communities (Weil, Losada & Bone, 1984; Weil, Torres & Ashton, 2005), densities could significantly vary in space and time. More recent observations made by checking mangrove root systems along 11 km in LPNR, found high densities of *Diadema* along the complex root system of exposed mangroves, with several localities having high densities of juveniles (<3 cm in diameter (E Weil, pers. obs., 1985). These complex habitats provide refuge and food and could become important recruitment and dispersal localities for *Diadema antillarum*.

Even though we occasionally find dying or recently dead urchins with similar signs to those of the disease of the early 1980’s, we have not observed any recent epizootic events causing significant urchin mortalities in south-west coast of Puerto Rico. With coral reefs currently facing an onslaught of anthropogenic and climate-induced stressors (Hughes, 1994; Hoegh-Guldberg et al., 2007; Jackson et al., 2014; Hughes et al., 2017), the derived benefits from the continued recovery of this keystone herbivore may aid to alleviate the impact of some of these stressors and facilitate recruitment and survivorship of foundation sessile species and reef recovery. Continuing to monitor *D. antillarum* and exploring the extrinsic factors contributing to the variable regional population recovery will provide valuable insight into the population dynamics of the sea urchin and the community composition and structure changes in Caribbean reefs over time.

## Conclusions

The long-spined sea urchin, *Diadema antillarum*, was once one of the most abundant keystone herbivores on Caribbean reefs until a species-specific epizootic event nearly eradicated its populations in the early 1980s. Following this event, many reefs throughout the region shifted from coral to macroalgal-dominated reefs. Recovery of *Diadema* populations have been very slow across the region. Results of this study indicate that *D. antillarum* densities vary spatially within and across reef localities in LPNR. Results also show that compared to 2001, urchin densities have slightly declined in LPRN, with population distributions mostly influenced by habitat complexity and food availability. A significant positive correlation was found between population density and habitat complexity; however, this correlation was weak, indication that habitat complexity is becoming less of a factor influencing urchin distributions on shallow reefs. Successful reproductive pulses and high movability could influence the densities and size structure observed in particular sites and times. Overall, *D. antillarum* populations continue to recover but at a slow and highly variable rate.

##  Supplemental Information

10.7717/peerj.8428/supp-1 Supplemental Information 1 Diadema raw density data

10.7717/peerj.8428/supp-2 Supplemental Information 2 Diadema oral test diameter raw data

## Additional Information and Declarations

### Data Availability

The following information was supplied regarding data availability: Raw numerical data is available in the Supplemental Files.
